# Detection of a genetic footprint of the sofosbuvir resistance-associated substitution S282T after HCV treatment failure

**DOI:** 10.1186/s12985-017-0779-4

**Published:** 2017-06-08

**Authors:** Andreas Walker, Sandra Filke, Nadine Lübke, Martin Obermeier, Rolf Kaiser, Dieter Häussinger, Jörg Timm, Hans H. Bock

**Affiliations:** 1 Institute for Virology, Heinrich-Heine-University, University Hospital, Universitätsstr. 1, 40225 Düsseldorf, Germany; 2 Department of Gastroenterology, Hepatology and Infectious Diseases, Heinrich-Heine-University, University Hospital, Düsseldorf, Germany; 3 Medical Center for Infectious Diseases (MIB), Berlin, Germany; 4 Institute of Virology, University of Cologne, Cologne, Germany

**Keywords:** Sofosbuvir, Daclatasvir, Resistance, Genotype 3a, Breakthrough, S282T, MEDCOAT

## Abstract

**Background:**

The major resistance-associated substitution for sofosbuvir (S282T) in HCV NS5B causes severe viral fitness costs and rapidly reverts back to prototype in the absence of selection pressure. Accordingly, resistance against sofosbuvir is rarely detected even in patients after treatment failure.

**Case presentation:**

We report a case of a GT3a infected patient with viral breakthrough under SOF/DCV therapy. At the time of breakthrough the RAS S282T was predominant in NS5B and then rapidly disappeared during follow-up by week 12 after treatment. Interestingly, despite only serine was encoded in position 282 during follow-up, two distinct genetic pathways for reversion were detectable. In 31% of the quasispecies the original codon for serine was present whereas in the majority of the quasispecies an alternative codon was selected. This alternative codon usage was unique for all GT3a isolates from the HCV database and remained detectable as a genetic footprint for prior resistance selection at the RNA level for at least 6 months.

**Conclusions:**

Comparative analyses of viral sequences at the codon level before and after DAA treatment may help to elucidate the patient’s history of resistance selection, which is particularly valuable for highly unfit substitutions that are detectable only for a short period of time. If such codon changes increase the risk of re-selection of resistance upon a second exposure to SOF remains to be addressed.

## Background

During the last few years, several directly acting antivirals (DAAs) became available for treatment of chronic hepatitis C, starting a new era of therapy of hepatitis C virus (HCV) infection. DAAs were typically optimized for inhibition of viral replication of HCV genotype 1a and 1b and are in most cases less active against genotype 3a. Currently, only a combination of the nucleoside analogue sofosbuvir (SOF) and one of the NS5A inhibitors Velpatasvir (VEL) or daclatasvir (DCV) are recommended for treatment of patients infected with HCV genotype 3a [1]. Depending on the fibrosis stage, prior treatment experience and the duration of therapy, sustained viral response rates (SVR) between 63 and 96% have been reported [2, 3]. In clinical studies resistance-associated substitutions (RAS) were detected in the majority of patients who failed to achieve SVR (reviewed in [4, 5]). In Genotype 3a the most common NS5A-RAS are Y93H, A30K and L31I [6]. In the ALLY-3 trial NS5A-Y93H RAS was present in 15 of 16 patients with relapse, whereas NS5B RASs associated with resistance to SOF (aa159, 282, or 321) were not detected [2]. Up to date the major SOF resistance associated substitution S282T was only found in few patients with viral relapse [7–9].

## Case presentation

**Fig. 1:**
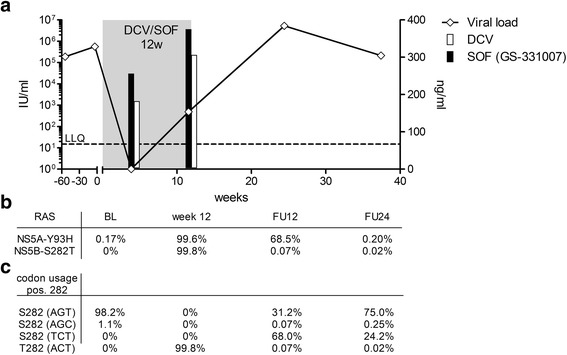
Therapy failure of a patient with HCV GT3a infection treated with SOF/DCV for 12 weeks. **a** HCV viral load (black line), time of treatment (gray box) and plasma drug levels for DCV (white bar) and the SOF metabolite GS-331007 (black bar) are shown. **b** Frequency of the RAS NS5A-Y93H and NS5B-S282T at baseline (BL), end of treatment (week 12) and follow-up (FU). **c** Codon usage at NS5B position 282 as determined by ultra-deep sequencing

We report here a case of a GT3a infected treatment naïve patient with viral breakthrough under SOF/DCV therapy. The patient was a 59 year old non-cirrhotic, HIV-negative woman treated at the University Hospital Düsseldorf, Germany. In accordance with the German and international guidelines treatment was initiated with 400 mg Sofosbuvir and 60 mg Daclatasvir once daily. Viral load at baseline was 562.530 IU/ml and dropped below the detection limit (<15 IU/ml) at week 4 (Fig. 1). Two days prior to the end of treatment at week 12 HCV-RNA was detectable again at low levels (493 IU/ml) consistent with a viral breakthrough. During follow-up 12 weeks after the end of treatment viral load further increased to high levels (5.290.000 IU/ml). Notably, due to difficulties with swallowing of tablets the patient had used MEDCOAT® tablet coating for assistance. Plasma drug levels at week 4 and at breakthrough were 182 ng/ml and 306 ng/ml for DCV and 256 ng/ml and 375 ng/ml for the SOF metabolite GS-331007 (Fig. 1). Amplicon sequencing [10] and resistance phenotyping using Geno2Pheno [HCV] (http://hcv.bioinf.mpi-inf.mpg.de/index.php; [11]) showed no RAS in NS5A and NS5B at baseline. Resistance testing at week 12 (day 82), when the patient was still under treatment, revealed the RAS S282T in NS5B and Y93H in NS5A (Fig. 1). Y93H persisted in 68.5% of the quasispecies during follow-up at week 12 and continued to revert back to nearly undetectable levels at follow-up at week 24. The substitution S282T had already fully reverted back to prototype at follow-up at week 12 consistent with severe fitness costs associated with this RAS. Interestingly, two distinct pathways were selected for reversion to the prototype residue. Ultra-deep sequencing revealed that 31% of the quasispecies reverted back to the original codon for serine (AGT) whereas in 68% of the quasispecies an alternative codon for serine (TCT) was selected. This alternative codon usage was unique and was absent from 114 GT3a isolates analyzed in our laboratory and from all 489 reference sequences from the HCV database. In line with negative selection of alternative codon usage in this position, the frequency of variants in the quasispecies utilizing the alternative codon further decreased to 24.2% during follow up at week 24.

## Discussion and conclusions

We report here, to our knowledge, the first case of selection of the RAS S282T in a patient infected with genotype GT3a. Selection of the RAS was associated with a viral breakthrough detected at week 12, when the plasma drug levels were in a low therapeutic range. If the low plasma drug levels are a consequence of the MEDCOAT® coating of the tablets is unknown since no peer reviewed data on the pharmacokinetics are available. However, it has been described that co-medication with proton pump inhibitors (PI) during HCV-treatment is a risk factor for treatment failure [12]. Accordingly, it seems plausible that the usage of MEDCOAT® might be a risk factor for treatment failure as well.

Although reversion of NS5A RAS has been described, in the majority (85–94%) of genotype 1 infected patients NS5A RAS persist in the viral population over years after treatment [13–15]. For genotype 3a no systematic analysis of the destiny of NS5A RAS after treatment failure is available. In line with previous reports the S282T substitution rapidly reverted back to prototype by week 12 after treatment [5]. Interestingly, despite only serine was encoded in position 282 at that time, the codon usage had changed leaving a detectable genetic footprint of prior resistance selection that was unique for GT3a isolates. This genetic footprint of prior resistance selection remained detectable at the RNA level for at least 6 months, consistent with lower fitness costs compared to the original RAS. We conclude that comparative analyses of viral sequences at the codon level before and after DAA treatment may help to elucidate the patient’s history of resistance selection, which is particularly valuable for highly unfit RAS that are detectable only for a short period of time. If such codon changes increase the risk of re-selection of resistance upon a second exposure to SOF is unknown. The patient reported here was not re-treated until now, as no advanced fibrosis was yet detectable.

## Abbreviations

DCV: Daclatasvir
HCV: Hepatitis C Virus
RAS: resistance-associate substitution
SOF: Sofosbuvir
